# Exploring the Antibacterial and Regenerative Properties of a Two-Stage Alginate Wound Dressing in a Rat Model of Purulent Wounds

**DOI:** 10.3390/biomedicines12092122

**Published:** 2024-09-18

**Authors:** Aliya Atepileva, Vyacheslav Ogay, Gulshahar Kudaibergen, Guldarigash Kaukabaeva, Assiya Nurkina, Ainur Mukhambetova, Serik Balgazarov, Arman Batpen, Dina Saginova, Zhanatay Ramazanov, Amanzhol Balgazarov, Zhanar Akhmetkarimova

**Affiliations:** 1 Department of Traumatology No. 4, National Scientific Center of Traumatology and Orthopedics Named after Academician N.D. Batpenov, Astana 010000, Kazakhstan; atepileva.nncto@mail.ru (A.A.); balgazarovss@gmail.com (S.B.); batpen_a@mail.ru (A.B.); saginova_d@nscto.kz (D.S.); 66zhanatai@hmail.com (Z.R.); amanzhol.balgazarov@gmail.com (A.B.); 2 Research School, Karaganda Medical University, Karaganda 100012, Kazakhstan; 3 Stem Cell Laboratory, National Center for Biotechnology, Astana 010000, Kazakhstan; ogay@biocenter.kz (V.O.); kudaibergen@biocenter.kz (G.K.); kaukabaeva@biocenter.kz (G.K.); nurkina@biocenter.kz (A.N.); mukhambetova@biocenter.kz (A.M.); 4 Toxicology and Pharmacology Laboratory, National Center for Biotechnology, Astana 010000, Kazakhstan

**Keywords:** alginate wound dressings, silver nanoparticles, cefepime, FGF-2, chronic wounds, antibacterial properties, regenerative medicine

## Abstract

Chronic wounds complicated by infection pose significant clinical challenges, necessitating comprehensive treatment approaches. The widespread use of antibiotics has led to resistant microorganisms, complicating traditional therapies. This study aims to develop and evaluate modified alginate wound dressings with enhanced antimicrobial and regenerative properties. Alginate dressings were synthesized with silver nanoparticles, cefepime, and fibroblast growth factor-2 (FGF-2). The two-stage therapy involved an initial antibacterial dressing followed by a regenerative dressing. In vitro tests demonstrated high antibacterial activity, with maximum inhibition zones for *P. aeruginosa* (41.3 ± 0.4 mm) and *S. aureus* (36.6 ± 1.8 mm). In vivo studies on rats with purulent wounds showed significant healing progression in the experimental group. Histological analysis revealed complete re-epithelialization, thicker neoepithelium, dense collagen deposition, and minimal inflammation in treated wounds. These findings suggest that the modified alginate dressings significantly enhance the reparative process and are promising for treating chronic infected wounds in both veterinary and medical practices.

## 1. Introduction

Chronic wounds represent a significant challenge in modern medicine, as they negatively impact patient quality of life and increase strain on healthcare systems. These wounds are frequently associated with serious complications, including sepsis, which results from persistent infection and inflammation in damaged tissues. The progression and severity of chronic wounds and their complications are influenced by the body’s defense mechanisms and the patient’s overall health, including factors such as diabetes, which impairs circulation and delays wound healing [1,2].

In chronic wounds, prolonged inflammation leads to the excessive production of reactive oxygen species (ROS) and enzymes. While ROS play a crucial role in defending against pathogens, their overproduction can lead to oxidative stress, resulting in damage to healthy tissues and further impairing the healing process [3,4,5]. In addition to the challenges posed by ROS and tissue damage, the rise of antibiotic-resistant microorganisms, driven by the overuse of antibiotics, further complicates wound healing. Low-level exposure to antibiotics, such as cefepime, in environmental settings has been shown to induce high-level resistance in bacteria, posing a significant public health risk [6,7,8,9]. These findings underscore the importance of developing wound treatments that limit antibiotic exposure to the broader environment. In the context of our alginate-based wound dressings, cefepime is applied in a localized manner, minimizing its potential release into the environment and reducing the risk of contributing to antibiotic resistance. Addressing these challenges requires the development of advanced wound dressings that combine both antimicrobial and regenerative properties [10,11,12,13,14]. Given the limitations of traditional therapies, innovative materials such as alginate, known for its ability to provide painless bandaging and promote gas exchange, were selected as the base for our dressing. However, alginate alone lacks sufficient structural integrity and does not possess intrinsic antimicrobial activity, necessitating enhancement through the incorporation of additional agents [14,15,16]. To this end, silver nanoparticles (AgNPs) and cefepime, a fourth-generation cephalosporin, were selected for their complementary antimicrobial properties. Silver nanoparticles are renowned for their broad-spectrum antimicrobial activity, which is effective against a wide range of pathogens, including antibiotic-resistant strains. Their multiple mechanisms of action—such as disrupting bacterial cell membranes, binding to microbial DNA, and generating ROS—make AgNPs a powerful tool in preventing infections [13,17,18,19]. However, the efficiency of AgNPs tends to decrease as bacterial concentration increases and exposure time shortens. To overcome this limitation, cefepime was coupled with AgNPs to enhance their effectiveness. Cefepime is a well-known broad-spectrum antibiotic, particularly effective against resistant bacteria, including Gram-negative strains, due to its stability against beta-lactamase enzymes [18,20,21]. We hypothesize that cefepime’s rapid and potent bactericidal action complements the slower, sustained activity of silver nanoparticles, providing an immediate response to infection while potentially reducing the risk of bacterial resistance. This combination results in enhanced overall antimicrobial efficacy [22]. This dual approach may offer both quick and long-lasting antimicrobial protection, which could be essential for managing the diverse microbial populations present in chronic wounds. Together, AgNPs and cefepime create a robust antimicrobial defense essential for managing infections in chronic wounds. However, while these agents are effective against infection, they do not significantly contribute to tissue regeneration. To address this limitation, fibroblast growth factor-2 (FGF-2) was incorporated into the dressing. FGF-2 is well documented for promoting angiogenesis, fibroblast proliferation, and granulation tissue formation, making it a key component in facilitating wound healing [3,23,24,25,26,27,28,29].

Recent studies have demonstrated the potential of combining multiple therapeutic functions in wound dressings. For instance, the Apollo-PAK-AM dressing incorporates antiseptics and anesthetics to control infection and reduce pain [30]. Moreover, Xilin Lin and colleagues demonstrated the benefits of a hydrogel dressing that integrates diclofenac and basic fibroblast growth factor (bFGF), resulting in enhanced healing outcomes [14]. These examples highlight the importance of multifunctional wound dressings that address both infection control and tissue repair. Fiblast, a commercially available product containing recombinant FGF-2, has shown promising results in accelerating wound healing. However, further innovation is required to develop dressings that integrate both antimicrobial and regenerative properties to fully meet the complex requirements of chronic wound care.

Based on this rationale, we developed a two-stage alginate wound dressing to address these challenges. The first stage, composed of alginate, AgNPs, and cefepime, targets infection, while the second stage incorporates AgNPs and FGF-2 to support tissue regeneration. This comprehensive strategy offers a promising solution for treating chronic infected wounds by providing both antimicrobial action and enhanced regenerative support

## 2. Materials and Methods

### 2.1. Animals

Male Wistar rats (10–12 weeks old, weighing 260–320 g) were purchased from the Pushchino laboratory animal breeding center (Pushchino, Russia). The rats were kept in a vivarium at a temperature of 23 °C and a relative humidity of 60%. The animals had free access to food and water. In vivo experiments were conducted in strict accordance with ethical standards for the treatment of animals, following the recommendations of the World Society for the Protection of Animals (WSPA) and the requirements of the European Convention for the Protection of Experimental Animals (Strasbourg, 1986). Additionally, these procedures were approved by the Local Ethical Committee for the Use of Animals at the National Center for Biotechnology.

### 2.2. Materials

In our studies, we used sodium alginate (W201502, Sigma-Aldrich, Steinheim am Albuch, Germany), 4-pentenoic anhydride, 98% (63521-92-6, Sigma-Aldrich, St. Louis, MO, USA), a broad-spectrum β-lactam cephalosporin antibiotic of the IV generation for parenteral use (A3737, Sigma-Aldrich, Steinheim am Albuch, Germany), and recombinant human FGF2 protein (Active, ab9596, Abcam, Cambridge, UK). *Staphylococcus aureus* ATCC 29213, a clinical isolate designated Wichita and utilized as a standard quality-control strain in laboratory testing, and *Pseudomonas aeruginosa* (Schroeter) Migula ATCC 27853 were purchased from the American Type Culture Collection (ATCC 27853). MTT assays (ab211091, Abcam, Cambridge, UK) were used to measure cell viability. Pristan (1921-70-6, Sigma-Aldrich, St. Louis, MO, USA) was used as an immunosuppressant because it slows down the tissue repair process. For cell diagnostics, histological, and clinical cytological examination of samples, Eosin Y Solution, alcoholic (HT110116, Sigma-Aldrich, St. Louis, MO, USA) was used for visualizing connective tissue with hematoxylin and eosin (H&E) staining.

### 2.3. Synthesis of Alginate Dressings

The alginate dressings were obtained in accordance with a previously described protocol [31]. Briefly, the hydrogel was obtained by modifying sodium alginate (Alg, 0.09 M) with 4-pentenoic anhydride (PA, 0.006 M) in 10 mL of Milli-Q (MQ) water with continuous stirring. The pH of the medium was maintained at 3 by adding concentrated acetic acid dropwise. The reaction temperature was 60 °C, and the duration was 6 h. The reaction product was precipitated with cold ethanol, then washed and lyophilized (freeze drying, Martin Christ Beta 2-8 LDplus). All procedures were performed in complete darkness. An Alg-PA solution with a concentration of 2.5% (*w*/*v*) was prepared in borosilicate vials using MQ water. I2959 (106797-53-9, Sigma-Aldrich, St. Louis, MO, USA) was added at a 0.5% concentration, after which the samples were exposed to UV light (365 nm) for one hour for click polymerization.

The mechanical strength of the hydrogels was determined by compressive testing using a texture analyzer (TA.XT plus, Version 6.1.4.0., Stable Micro System, Godalming, UK). Cylindrical samples were prepared with a diameter of 10 mm and a height of 5 mm. Compression tests were performed at a constant strain rate until the hydrogel samples deformed or ruptured. The compressive strength was calculated by dividing the maximum force at failure by the cross-sectional area of the sample. The stress–strain curves were recorded, and the maximum compressive strength values were obtained for each sample.

The density of the hydrogel, gel fraction, and degradation rate were assessed using standard methods. The gel yield was calculated using the following formula:

(1) $$Gel\;(\%)=\frac{W_{d}}{W_{s}}\times 100,$$

where $W_{d}$ and $W_{s}$ are the masses of the dry and swollen samples, respectively. The hydrogel was placed in sterile phosphate-buffered saline (PBS) (0.1 M, pH 7.4) at 37 °C for eight weeks, with the solution being replaced twice a week. After lyophilization (resulting in samples $W_{2}$), the degradation rate was calculated using the following formula:

(2) $$DD\;(\%)=\frac{W_{1}-W_{2}}{W_{1}}\times 100,$$

The swelling process was evaluated by the gravimetric method, determining the swelling degree by mass as follows:

(3) $$\alpha \;(\%)=\frac{m_{n}-m_{1}}{m_{1}}\times 100,$$

where $m_{1}$ is the initial mass, and $m_{n}$ is the mass after a certain period of time.

Silver, cefepime, and FGF-2 were incorporated into the alginate structure by absorption. The lyophilized hydrogel was immersed in a combined solution of 0.1 M silver nitrate and 0.5 M cefepime for 24 h. Following absorption, the hydrogel was rinsed with deionized water to remove any unabsorbed substances. To absorb FGF-2, the hydrogel was incubated in an FGF-2 solution (concentration 50 µg/mL) for 12 h at 4 °C, then rinsed with PBS. The control hydrogel, used specifically to study FGF-2 activity, was prepared from sodium alginate and calcium chloride, with FGF-2 particles absorbed (Alg-Ca-FGF-2), but without the addition of silver or cefepime.

### 2.4. Nuclear Magnetic Resonance

Solutions of alginate and its derivatives (1% *w*/*v*) were prepared in D_2_O, and 4-pentenoic anhydride (1% *w*/*v*) in CD_3_Cl. ^1^H NMR spectra of the samples were recorded using a JNM-ECA 500 spectrometer (JEOL, Peabody, MA, USA). MestreNova software (Version 6.0.2-5475) was used for spectral data processing. The degree of substitution of PA in the main chain of Alg was assessed by analyzing the ^1^H NMR spectra. The percentage degree of substitution of PA (%DM) in the Alg chain was determined using the following equation [31]:

(4) $$\%DM=\frac{H_{b}+H_{C}/2}{GG-1/6}\times 100,$$

where, ${H_{C},\;H}_{b}$, and GG-1 represent the hydrogen signals of the anomeric carbon in mannuronic units, associated peaks, and hydrogens of the pentenoic group, respectively.

### 2.5. Scanning Electron Microscopy

The hydrogel samples were dehydrated using a Martin Christ Beta 2-8 LD plus lyophilizer (Martin Christ Gefriertrocknungsanlagen GmbH, Osterode am Harz, Germany). The morphology of the lyophilized hydrogel was then analyzed using a scanning electron microscope (SEM, Auriga Crossbeam 540, Carl Zeiss, Oberkochen, Germany) after being coated with a 10 nm thick layer of gold. The SEM operated in SE2 signal mode, set to an analytical column, with a working distance (WD) of 4.6 mm, Line Int. Busy SB Grid 833 V noise reduction, and a scan speed of 3.

### 2.6. FGF-2 Release Kinetics

The release of the FGF-2 was carried out using an enzyme-linked immunosorbent assay (ELISA). The release kinetics of FGF-2 from the alginate hydrogels were studied using a sandwich ELISA. The alginate hydrogel containing 100 ng/mL FGF-2 was placed in microcentrifuge tubes (2 mL) and incubated in 1 mL of PBS at 37 °C in a thermostat with constant stirring in a vortex. Every 24 h of incubation, the supernatant was collected and replaced with 1 mL of fresh PBS. The amount of FGF-2 released from the alginate hydrogel was determined using commercial ELISA kits (RAB0182, Sigma, Dorset, UK).

### 2.7. MTT Assay

The level of cell viability and proliferation was determined using an MTT assay kit (Abcam, USA), according to the manufacturer’s instructions. Briefly, cells were seeded into 96-well plates and centrifuged at 1000× *g* and 4 °C for 5 min. The medium was removed, and 50 µL of serum-free medium and 50 µL of MTT reagent were added to each well. Control wells contained only the MTT reagent and medium. The plate was incubated at 37 °C for 3 h. Then, 150 µL of MTT solvent was added to each well, the plate was shaken for 15 min, and the absorbance was measured at OD = 590 nm. Incubation of the samples was carried out for 24, 48, and 72 h and 1, 7, and 14 days. Reagents were brought to room temperature before use, aliquoted into working volumes, and then stored at −20 °C, protected from light. Bubbles and foam formation were avoided when handling reagents, new tips were used for each addition, and all biological materials were disposed of according to established safety procedure.

### 2.8. Analysis of Antibacterial Activity

To determine the cell titer, the commercial strains *S. aureus* and *P. aeruginosa* were cultured overnight at 37 °C, 150 rpm, in 5 mL of LB broth. An amount of 1 mL of the overnight culture was diluted in 9 mL of NaCl solution and 100 µL was plated onto LB agar Petri dishes. The colony-forming units (CFUs) in 0.1 mL of the diluted culture were determined using the serial dilution method. The results were averaged to determine the number of colonies grown from the inoculation of each dilution. For reliable results, plates with a bacterial colony count ranging from 30 to 300 were selected. The average number of CFUs (N) per 1 mL was calculated using the following formula:

(5) $$N=\frac{c}{\left(n_{1}+0.1\times n_{2}\right)\times d},$$

where

*c* is the sum of the counted colonies on all plates;
$n_{1}$ is the number of plates of the first dilution;
$n_{2}$ is the number of plates of the second dilution;
d is the coefficient of the first dilution;
0.1 is the coefficient accounting for the dilution factor of both the first and second dilutions.

The cell titer was determined by the following formula:

(6) $$\sum x\frac{CFU}{mL},$$

where $x\frac{CFU}{mL}$ is the sum of colony-forming units in 0.1 mL of the diluted culture.

Thus, the titer of cells of the overnight cultures of *S. aureus* and *P. aeruginosa* was 11.6 × 10^9^ cells/0.1 mL and 22.4 × 10^9^ cells/0.1 mL.

Briefly, the antibacterial activity of the alginate dressings was evaluated using a well diffusion method similar to the protocol described by Zafar et al. [32]. Nutrient agar plates were prepared with four wells, and a 100 µL bacterial suspension of *S. aureus* and *E. coli* was spread evenly across the surface. Low, medium, and high doses of Ag NPs and cefepime solutions were introduced into the wells. The plates were incubated at 37 °C for 24 h. After incubation, the diameters of the inhibition zones around each well were measured with precision. The results were recorded as the mean and standard deviation (SD) from three replicates for each strain.

### 2.9. Modeling a Purulent Wound in Rats (Location Selection)

The induction of pathology and surgical intervention to create skin wounds were performed under anesthesia. Isoflurane was used as the anesthetic, initially at 5% in an induction chamber with an oxygen flow rate of 2–3 L per minute, then at 2% via a face mask, not exceeding 4% to avoid rapid overdose. Before creating full-thickness wounds, the fur in the surgical field area was trimmed with scissors. Residual fur was removed using depilatory cream (Eveline Cosmetics, Warsaw, Poland), which was applied for 3 min. The surgical field was then sequentially treated once with a 5% alcoholic iodine solution and 70% ethanol. Using a sterile 8 mm Dermo-punch biopsy stiletto (Sterylab, Milano, Italy), full-thickness skin wounds extending to the superficial muscle fascia were created on the rats through a stretched skin fold. The excised skin was removed with tweezers and scissors, and a 2 mm thick silicone ring with a 10 mm inner diameter hole was sutured in place (suture material Vicryl 3-0). The location selection for the purulent wound was investigated in two groups (n = 3):

1. Experimental animals with purulent wound modeling (*S. aureus* + *P. aeruginosa*) with wounds created in the withers area.
2. Experimental animals with purulent wound modeling (*S. aureus* + *P. aeruginosa*) with wounds created in the femoral–gluteal area.

### 2.10. Evaluation of the Safety and Efficacy of Two-Stage Therapy Using Alginate Dressings in Rats

After immunosuppression of the animals, wounds were modeled, and a bacterial mixture was added. Pristan (Sigma-Aldrich, USA) was used as the immunosuppressant. The animals with the purulent wound model were divided into four groups of five rats each, depending on the type of wound dressing used:

1. Experimental animals with purulent wound modeling (*S. aureus* + *P. aeruginosa*) using alginate dressings absorbed with silver particles;
2. Experimental animals with purulent wound modeling (*S. aureus* + *P. aeruginosa*) using alginate dressings absorbed with the antibiotic cefepime;
3. Experimental animals with purulent wound modeling (*S. aureus* + *P. aeruginosa*) using alginate dressings absorbed with silver particles and the antibiotic cefepime;
4. Experimental animals with purulent wound modeling (*S. aureus* + *P. aeruginosa*) using the traditional method of treating purulent wounds—daily dressings with aseptic solutions and ointments.

Two-stage alginate dressing therapy involves a hydrogel with antibacterial and regenerative activity. In the first stage, dressings with silver, cefepime, and their combination (silver and cefepime) were used. After reducing the bacterial load of the wound, the second stage of healing was applied using an alginate dressing with FGF-2. Over the course of two weeks, the wound surface area, results of bacterial cultures, and the presence or absence of pus were evaluated.

### 2.11. Histological Analysis

All tissue samples from purulent wounds were fixed in 10% neutral buffered formalin and decalcified using an electrolytic decalcifying solution for three days (BioVitrum, Saint Petersburg, Russia). After the dehydration process, the samples were embedded in paraffin and sectioned into 5 μm thick slices. The cross-sections were stained with 1% Eosin Y Solution, alcoholic (Sigma-Aldrich, USA). The stained sections were examined at 70×, 120×, and 400× magnification using an Axio Scope A1 upright microscope (Carl Zeiss, Oberkochen, Germany) to measure the formation of new epidermal tissue in the defect area. All obtained images were processed using ImageJ software (version 1.53t, NIH, Bethesda, MD, USA).

### 2.12. Statistical Analysis

All data are presented as mean ± standard deviation (SD). Statistical significance was verified using one-way analysis of variance (ANOVA) followed by Bonferroni multiple comparison tests. A value of *p* < 0.05 was considered statistically significant. Quantitative data are presented as mean ± standard error (SE). The Statistica 6.0 program (StatSoft, Tulsa, OK, USA) was used for data analysis.

## 3. Results

### 3.1. Characterization of Alginate Hydrogel

**Figure 1:**
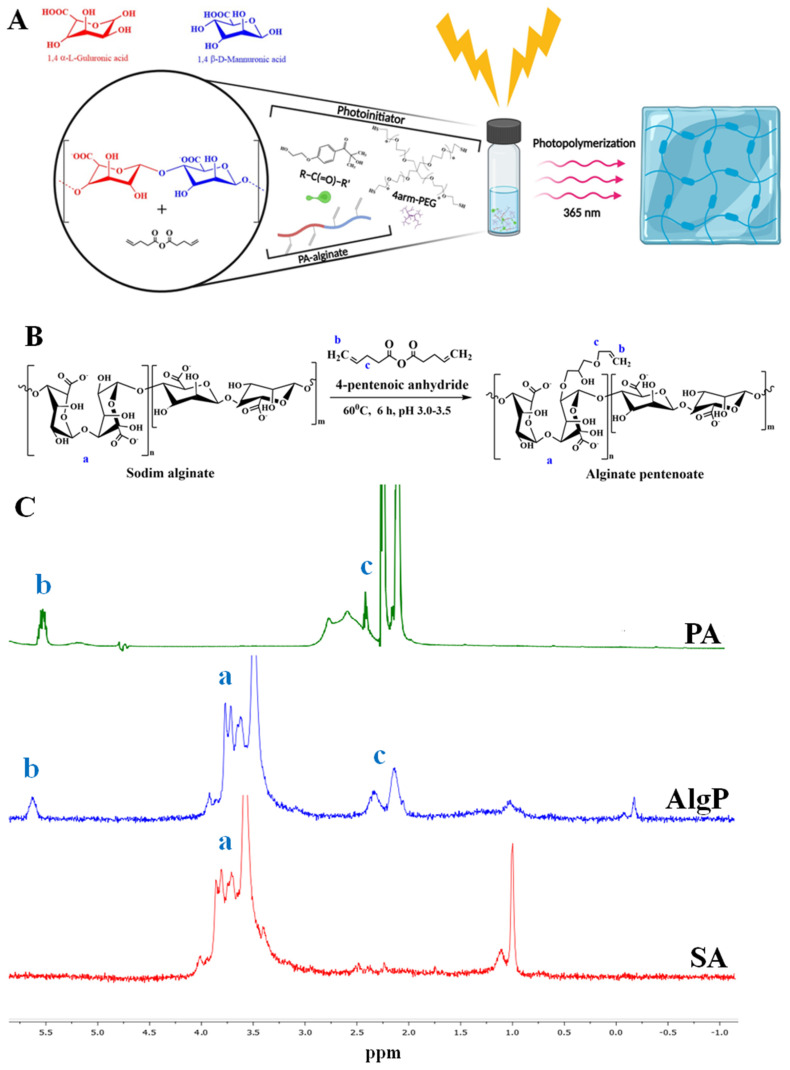

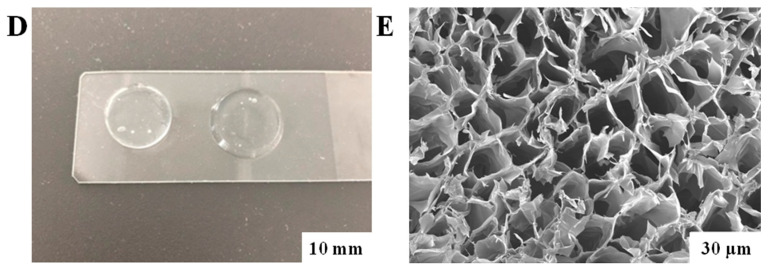
Synthesis and characterization of alginate hydrogel. (**A**) General graphical illustration of the synthesis process for the modified alginate hydrogel. (**B**) Schematic illustration of the sodium alginate hydrogel modification. Please note that the schematic structure shows only one possible pathway of the click chemistry reaction. In reality, the reaction could occur with any hydroxyl groups present in the sodium alginate. (**C**) ^1^H NMR spectra of sodium alginate (Alg), 4—pentenoic anhydride (PA), and their resulting product (AlgP) recorded in D_2_O at 50 °C. (**D**) Illustration of the alginate hydrogel disk. (**E**) SEM image of the surface morphology of the alginate hydrogel. Scale bar: 30 µm.

In this study, a sodium alginate solution (SA, 0.09 M) was prepared in 10 mL of Milli-Q water for the synthesis of modified sodium alginate. While continuously stirring, pentenoic anhydride (PA, 0.006 M) was added to the alginate solution. The reaction was carried out in an acidic medium (pH = 3) with the addition of concentrated acetic acid at 60 °C for six hours. A large amount of cold ethanol was added to precipitate the product. After the reaction, the product was precipitated using a large volume of cold ethanol, washed 2–3 times with ethanol, and then dried via lyophilization. The entire synthesis process was carried out in complete darkness. Modified alginate was synthesized by the reaction of sodium alginate with pentenoic anhydride (Figure 1A). After dialysis purification, the structure of the resulting alginate matrix was examined by ^1^H NMR spectroscopy (Figure 1B,C). The spectrum showed characteristic peaks confirming the successful introduction of 4-pentenoic groups into the alginate structure (AlgP). Two singlets were observed in the regions 2.5–2.3 ppm (c) and 2.3–2.1 ppm (c), corresponding to the protons of the OOCCH_2_CH_2_CH=CH_2_ group. The signals of the protons of the CH and CH_2_ groups of the glucose part of the alginate molecule appeared in the region of 3.2–3.9 ppm (a) as a complex multiplet, which is typical for this structure. The methylene protons of the vinyl fragment CH=CH_2_ gave a singlet in the region of 5.5–5.7 ppm (b), confirming the presence of vinyl groups in the modified product. Minor impurities were observed at −0.25 ppm and 1 ppm in the spectrum. These peaks likely correspond to trace residual solvents or unreacted small molecules, but they are present at very low intensities and do not affect the overall structure or integrity of the modified alginate product. Additionally, the characteristic peaks clearly show that the intended modifications were successfully made to the alginate. The integral curve of the spectrum corresponded to the total number of protons, indicating the correct ratio between the various groups in the structure of the modified alginate (Figure 1C).

The tensile strength of the modified alginate hydrogel increased from 320 kPa to 420 kPa, indicating a substantial enhancement in its mechanical properties. This improvement is attributed to the chemical cross-linking process. The sol–gel content is an important parameter as it reflects the proportion of the insoluble and soluble fractions in the polymer, affecting its properties. The average gel content in the studied samples is 71–73%, indicating a high proportion of the insoluble fraction (Figure 1D). The swelling of hydrogels is characterized by two main processes: solvation of the pore walls and their filling with solvent, leading to an increase in pores and solvent absorption [33]. The high degree of swelling of the alginate hydrogel over 24 h (28.7 ± 1.4%) indicates its significant capacity for fluid absorption. This property is important for use in wound dressings where high exudate absorption is necessary. Crosslinking of thiol-ene groups likely enhances the mechanical strength of the pore walls, preventing their collapse and ensuring the stability of the hydrogel structure. Morphological studies confirmed that the freeze-drying process does not alter the pore structure, which is important for preserving the properties of the hydrogel [31].

The overall morphology of the alginate hydrogel after the gelation process, as well as the surface morphology examined using SEM, is shown in Figure 1E. Surface morphology studies of the polymers showed that a porous structure forms in the samples. The hydrogel demonstrated an average pore size of 27.0 ± 10 μm. Notably, the swelling capacity of the hydrogel is consistent with the previously observed phenomenon that it depends on the pore size of the polymer. Larger pores facilitate increased fluid absorption by the polymer [33]. Moreover, it is known that the presence of larger pores enhances cell proliferation and adhesion. In addition, the pores provide intercellular connectivity and interaction with silver and cefepime particles, making the hydrogel a promising medical device in regenerative medicine.

**Figure 2:**
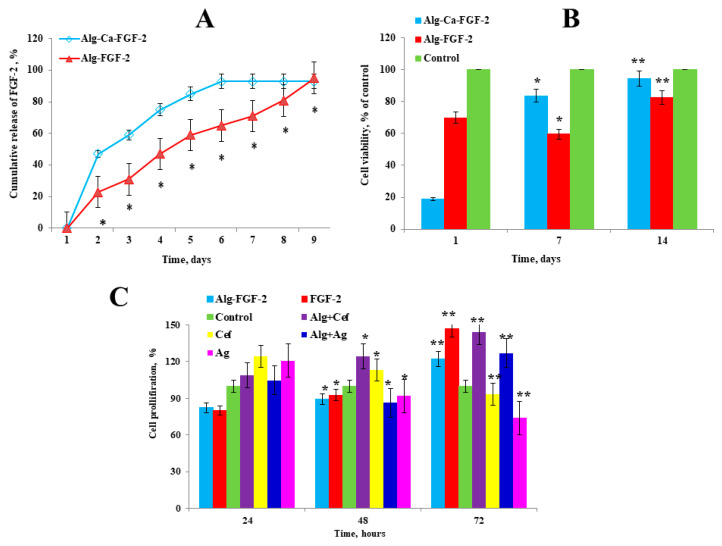
Evaluation of FGF-2 release and cytotoxicity of alginate hydrogels. (**A**) Profile of cumulative FGF-2 release from Alg-Ca-FGF-2 and Alg-FGF-2 hydrogels. (**B**) MTT assay for cytotoxicity of alginate hydrogels on rat ADMSC cells. (**C**) The effect of dressings with FGF-2, cefepime, and silver nanoparticles on the growth of dermal fibroblasts. Positive controls: FGF-2 (100 ng/mL), cefepime (10 mg/mL), and AgNPs (10 mg/mL) in culture medium. Values are presented as the mean value ± SD (n = 5). * *p* < 0.05, ** *p* < 0.01.

We evaluated the kinetics of the release of FGF-2 from alginate hydrogel using an ELISA. The profile of FGF-2 release from the alginate hydrogel is shown in Figure 2A. The ELISA demonstrated a burst release of 47.2 ± 1.8% of the total quantity of FGF-2 from the Alg-Ca-FGF-2 hydrogel within 2 days, followed by 90.4 ± 2.4% of the total quantity of FGF-2 after 10 days of incubation in PBS. In contrast, the release of FGF-2 from the Alg-FGF-2 hydrogel was significantly more stable. Within 2 days, 23.1 ± 1.2% of the total quantity of FGF-2 was released from the alginate hydrogel, and 71.1 ± 3.3% of FGF-2 was detected on day 10. The complete release of FGF-2 from the Alg-FGF-2 hydrogel was observed on day 14. Thus, our results indicate that the Alg-FGF-2 hydrogel demonstrated a controlled release of fibroblast growth factor-2 in a sustained manner.

We conducted a toxicity test (Figure 2B), where the percentage of cell viability was determined by calculating the ratio of optical density (ODexp) in the experimental group to the optical density (ODcontr) in the control group in accordance with the international standard ISO 10993-5:2009(E) [34]. According to this standard, cytotoxicity is divided into six degrees, 0, 1, 2, 3, 4, and 5, corresponding to 100%, 75–99%, 50–74%, 25–49%, 1–24%, and 0% cell viability, respectively, with degrees 0 and 1 considered non-cytotoxic. The same initial cell density was seeded for all samples. On day 1, a decline in cell viability was observed in the Alg-FGF-2 group, possibly due to an initial stress response or a delayed release of FGF-2 from the alginate matrix. However, by day 7, a significant increase in cell proliferation was noted, suggesting that the sustained release of FGF-2 from the dressing effectively promoted cell growth over time. The cell viability rate for the Alg-FGF-2 hydrogel was 94.5 ± 0.6%, while the Alg-Ca-FGF-2 hydrogel showed 82.4 ± 0.5% viability. Both hydrogels indicated the absence of cytotoxicity as they were above the 80% threshold (Figure 2B).

Further, we performed an MTT assay to assess proliferation at various stages of incubation with hydrogels (Figure 2C). After 24 h, the highest cell viability was observed in wells with cefepime (124.3 ± 0.5%), indicating a strong early stimulatory effect. Alg+Cef also showed increased viability at 108.7 ± 0.5%, while Alg-FGF-2 and Alg+Ag demonstrated moderate effects with 82.3 ± 0.5% and 104.5 ± 0.5%, respectively. Ag alone increased cell viability to 120.5 ± 0.5%, while control cells maintained 100% viability. After 48 h, the highest cell viability was observed in wells with FGF-2 (92.4 ± 0.4%), indicating its continued positive effect on cellular proliferation. Alg+Cef also showed a significant increase in cell viability to 124.3 ± 0.6%, while Alg-FGF-2 maintained a high viability of 89.3 ± 0.5%. In contrast, Alg+Ag decreased to 86.9 ± 0.6%, and Ag alone reduced slightly to 91.9 ± 0.5%, indicating a reduction in their effect over time. At 72 h, FGF-2 and Alg+Cef showed the highest cell viabilities, reaching 147.0 ± 0.5% and 143.7 ± 0.6%, respectively, suggesting that their stimulatory effects peak after extended incubation. Alg-FGF-2 increased to 121.9 ± 0.5%, while Alg+Ag maintained a strong viability at 126.9 ± 0.6%. However, Cef and Ag alone showed reduced effects over time, with viabilities of 93.1 ± 0.5% and 73.8 ± 0.5%, respectively. These results indicate that while FGF-2, Alg+Cef, and Alg+Ag promote cell proliferation, further investigation is necessary to better understand the mechanisms behind the observed decreases in effectiveness for Cef, Ag, and FGF-2 over time. This will help optimize their use in hydrogel formulations for improved therapeutic outcomes.

**Figure 3:**
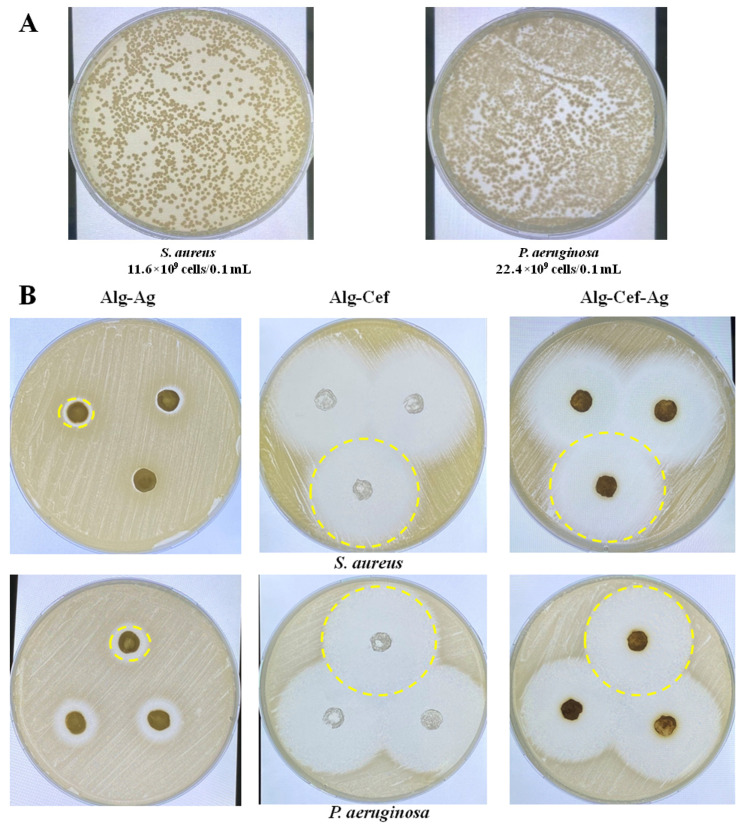
Antibacterial activity of alginate hydrogels. (**A**) Titer of cells from the overnight cultures of *S. aureus* and *P. aeruginosa* determined by visual counting of colonies after inoculation on LB culture medium. (**B**) Inhibition zones of test cultures of *S. aureus* and *P. aeruginosa* after incubation with hydrogel samples containing silver particles and cefepime at a concentration of 1%. The values are presented as the mean value ± SD (n = 5).

The next step was to determine the titer of overnight cultures of *Staphylococcus aureus* and *Pseudomonas aeruginosa*. The percentage of viable cells was visually calculated by counting the number of colonies grown after inoculation of microorganisms on LB nutrient media. The titer was 11.6 × 10^9^ CFU/0.1 mL for *S. aureus* and 22.4 × 10^9^ CFU/0.1 mL for *P. aeruginosa* (Figure 3A). A microbiological disk diffusion method was used to evaluate the antibacterial properties of alginate hydrogels (Figure 3B and Table 1). This method measures the diameter of the inhibition zones, which allows for a practical assessment of antimicrobial efficacy, particularly for silver nanoparticles, as shown in previous studies [32,35]. Test cultures of *S. aureus* and *P. aeruginosa* were incubated with hydrogel samples containing silver particles and cefepime at a concentration of 1%. The hydrogels were presterilized with a UV lamp for 15 min. The results showed that hydrogels with Alg-Cef and Alg-Cef-Ag demonstrated significant antibacterial activity. For *P. aeruginosa*, the largest inhibition zone was observed in hydrogels with Alg-Cef-Ag (41.3 ± 0.4 mm) and Alg-Cef (38.1 ± 0.9 mm). For *S. aureus*, the most effective hydrogels were Alg-Cef-Ag (36.6 ± 1.8 mm) and Alg-Cef (30.1 ± 0.5 mm). Alg-Ag samples demonstrated lower antibacterial activity: 3.5 mm for *P. aeruginosa* and 2.0 mm for *S. aureus*, respectively. However, despite the lower values, the Alg-Ag hydrogel still exhibited antibacterial properties. These results confirm that alginate hydrogels with the addition of silver and cefepime demonstrate a synergistic effect, in which their combined action significantly exceeds the sum of each component’s individual effect. Additionally, such hydrogels support cell viability and ensure the controlled release of growth factors, making them promising materials for use in regenerative medicine and the treatment of infectious diseases.

### 3.2. Evaluation of the Safety and Efficacy of the Two-Stage Dressing in Rats

**Figure 4:**
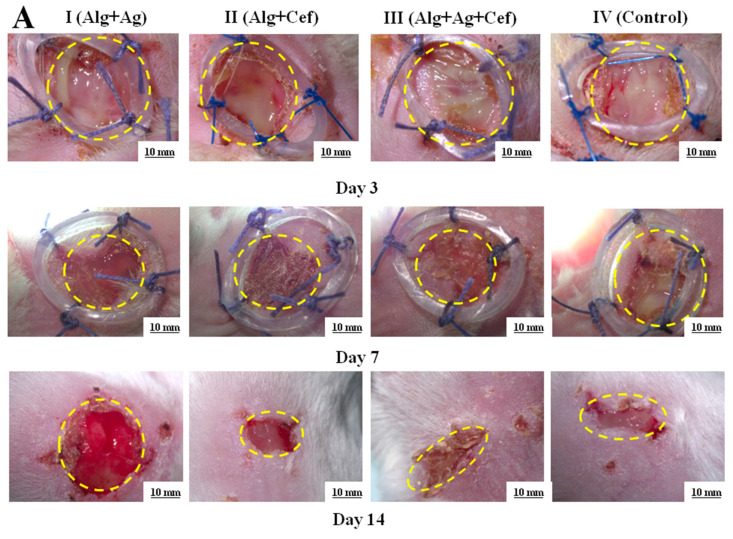

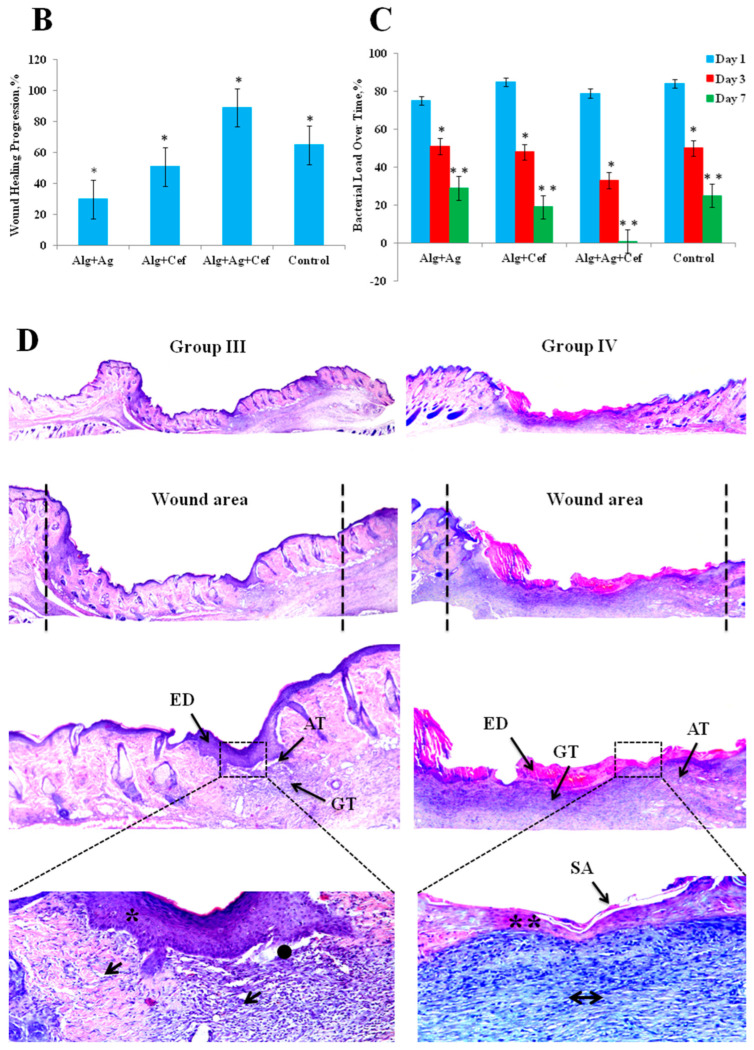
Regeneration of epidermal tissue in purulent wounds of rats using two–stage alginate dressing therapy. (**A**) Microscopic images of purulent wounds at 3, 7, and 14 days. (**B**) Wound healing rates in control and experimental groups. (**C**) Results of bacterial cultures taken from wounds on days 1, 3, and 7. * *p* < 0.05, ** *p* < 0.01. (**D**) Histological image of a wound area section: ED—epidermis; GT—granulation tissue; AT—adipose tissue; SA—skin appendage; robust, wellrevascularized GT (↓); poorly developed GT (↔); well—formed, dense ED (*); thin, fragile ED (**); regenerated AT (●).

The results of the study indicated that the wound healing rates in rats of groups I (Alg+Ag) and II (Alg+Cef) were almost identical throughout the experiment. On the 7th day, the wound surface area was around 80% of the initial wound size, and by the 14th day, it had reduced to 70% (Figure 4A,B). In group II, no microorganism growth was detected in the bacterial cultures on the seventh day, whereas in group I, microorganisms were found (Figure 4C). In group III (Alg+Ag+Cef), pus formation was observed on the 3rd day, and by the 7th day, the wound healing was at 55% of the initial wound area, with no microorganisms detected in the bacterial cultures (Figure 4C). In the control group (IV), pus formation persisted until day 10, with microorganisms found in the bacterial cultures. Wound healing in this group began only on the 14th day. Alginate dressings with FGF-2 were used in the second stage of therapy after the bactericidal load had been reduced. It is noteworthy that the application of alginate dressings with FGF-2 significantly promoted tissue regeneration in all experimental groups compared to the control group. Consequently, alginate dressings with FGF-2 demonstrate high regenerative potential.

Histological analysis was performed on animals from groups III and IV (Figure 4D), as the wound healing rates in rats of groups I and II were nearly identical and remained below 70% throughout the experiment.

In group III, complete re-epithelialization of the wound defect area with more than 95% closure was observed. The thickness of the newly formed epidermis was generally higher than the average thickness of the epidermis in intact skin (Figure 4D*). In some sections, weakly attached or lost keratin layers, or a thick parakeratotic stratum corneum, were noted (Figure 4D↓). In the dermis, granulation tissue and collagen deposition were observed, primarily characterized by a mixed fiber structure comprising filamentous fibers of both immature and mature collagen. Partial remodeling of the dermis was also evident, including collagen deposition and partial restoration of dermal white adipose tissue (Figure 4D●). In group IV, partial re-epithelialization of the wound defect area with less than 95% closure was observed. In the dermis, there was granulation tissue and collagen deposition (Figure 4D**), which predominantly had a reticular fiber structure represented by filamentous fibers of immature collagen (Figure 4D↔). Partial remodeling of the dermis was also noted, including collagen deposition and partial restoration of dermal white adipose tissue.

## 4. Discussion

This study explored the development and efficacy of a novel two-stage alginate wound dressing incorporating silver nanoparticles (AgNPs), cefepime, and fibroblast growth factor-2 (FGF-2) to treat chronic infected wounds. Given the increasing challenge of antibiotic resistance, this approach aims to address the dual needs of infection control and tissue regeneration. Our findings align with current research, demonstrating that combining antimicrobial and regenerative therapies in a single dressing can significantly improve wound healing outcomes [15,16,17].

Using click chemistry, we modified the alginate polymer by functionalizing it with 4-pentenoic anhydride, resulting in enhanced mechanical strength and improved physicochemical properties. The modified alginate hydrogel achieved a mechanical strength of 420 kPa, which aligns with similar studies where chemical cross-linking significantly enhances structural integrity, making it suitable for advanced biomedical applications [36,37]. In addition to its mechanical improvements, the modified alginate exhibited a gel content of 71–73% and a swelling capacity of 28.7 ± 1.4%, crucial for maintaining a moist wound environment and supporting structural stability during healing. SEM analysis revealed increased porosity, which promotes cell proliferation and adhesion, aiding in tissue regeneration. These characteristics, alongside a favorable ash content and a controlled degradation rate, confirm the hydrogel’s suitability as a wound dressing material, consistent with findings by Zhang et al. [15].

One of the primary innovations in this study was the controlled release of FGF-2. The initial burst release of 47.2 ± 1.8% within the first two days, followed by sustained release over 10 days, provided continuous support for early angiogenesis and fibroblast proliferation, which are critical for wound repair. This aligns with the findings of Dinh et al. [27] and Han et al. [23], who emphasize the role of growth factors in accelerating tissue regeneration. However, after 24 h, a decrease in FGF-2 efficacy was observed, which may be due to the natural degradation or inactivation of the growth factor over time, as well as cellular saturation with FGF-2’s mitogenic effects. Similarly, the reduction in cell viability observed with silver and cefepime toward the final day is likely related to their antimicrobial mechanisms. While they show strong initial antibacterial activity, prolonged exposure could induce cytotoxic effects, reducing cell viability over time. Additionally, their release profiles may taper off after an early peak, leading to diminished effectiveness by the 72 h mark. Our cytotoxicity assays confirmed that the hydrogels were biocompatible, with cell viability rates of 94.5 ± 0.6%, supporting the safety of the dressing for clinical use. Optimizing the release profiles of FGF-2, silver, and cefepime will be crucial to maintaining their efficacy over extended periods while minimizing any negative impact on cell viability.

The combination of AgNPs and cefepime within the alginate matrix demonstrated superior antibacterial efficacy, producing larger inhibition zones against *Pseudomonas aeruginosa* (41.3 ± 0.4 mm) and *Staphylococcus aureus* (36.6 ± 1.8 mm) compared to single-agent treatments. This synergistic effect echoes the findings of Bruna et al. [38], who demonstrated that combining antibiotics with nanoparticles enhances antibacterial effectiveness, particularly against resistant strains. In vivo, the dual-agent dressing accelerated wound closure and reduced purulent discharge, proving more effective than traditional methods. Thus, the integration of AgNPs and cefepime provides robust, dual-layered protection against infection, ensuring more efficient wound management.

In our in vivo studies, we simulated purulent inflammation in laboratory animals, focusing on two anatomical regions: the gluteofemoral area and the withers. This allowed us to compare healing responses and evaluate the practical application of the dressing in veterinary medicine. In the gluteofemoral wound group, 30% of the animals exhibited suture licking within 48 h, which resulted in wound sanitation and reduced inflammation. By 72 h, 50% of the animals showed mild hyperemia and swelling, with some purulent discharge. In contrast, while no suture licking was observed in the withers wound group at 48 h, 95% of the animals still developed significant hyperemia, edema, and purulent discharge by 72 h. The withers region proved more suitable for controlled studies due to its protection from external contamination [39]. Histological analysis further confirmed the regenerative potential of the two-stage alginate dressing. Animals treated with the dual-agent dressing exhibited well-organized epidermal layers, dense collagen deposition, and adipose tissue formation beneath the dermis, indicating comprehensive tissue regeneration. Conversely, the control group showed persistent inflammation and incomplete healing. These findings align with the study by Lin et al. [31], which demonstrated that staged drug delivery systems provide superior regenerative outcomes compared to single-phase treatments.

Overall, this study demonstrates that the dual-action mechanism of combining antimicrobial and regenerative therapies within a single dressing offers substantial advantages. The staged release of cefepime and FGF-2 mirrors the findings of Boateng and Catanzano [17], who reported that multifunctional dressings improve healing outcomes by addressing both infection and regeneration in a coordinated manner. However, limitations such as the cost of growth factors and the risk of allergic reactions, as noted by Dolati et al. [24], highlight areas for future optimization. In conclusion, while the dressing offers a significant therapeutic advantage, future research should focus on making it more cost-effective and minimizing potential side effects.

## 5. Conclusions

The two-stage alginate dressing developed in this study shows strong potential as an effective treatment for chronic infected wounds by combining antimicrobial action with regenerative support. Histological analysis revealed enhanced healing compared to standard treatments, with complete re-epithelialization and the formation of more mature and organized tissue. Based on these promising results, we plan to carry out further studies to optimize the release profiles and expand the use of this dressing in both clinical and veterinary applications.

## Figures and Tables

**Table 1:** Antibacterial efficacy of alginate hydrogels against *P. aeruginosa* and *S. aureus*.

Strains/Samples	Alg-Ag	Alg-Ag-Cef	Alg-Cef
*P. aeruginosa*	3.5 ± 0.5	41.3 ± 0.4	38.1 ± 0.9
*S. aureus*	2.0 ± 0.4	36.6 ± 1.8	30.1 ± 0.5

Inhibition zones measured in millimeters (mm). Values are presented as mean ± standard deviation.

## Data Availability

All data generated or analyzed during this study are included in this article.

## References

[1] Shmatenko O.P., Pidlisny O.V., Prykhodko T.V., Solomennyi A.M., Pritula R.L., Semenchenko G.B., Takhtaulova N.O. (2020). Technological aspects of creating soft dosage forms for the treatment of purulent wounds. Ukr. J. Mil. Med..

[2] Zubarev P.N., Ivanus S.Y., Risman B. (2017). V Modern Principles of Treatment of Purulent Wounds.

[3] Guo S., Dipietro L.A. (2010). Factors Affecting Wound Healing. J. Dent. Res..

[4] Medzhitov R. (2008). Origin and Physiological Roles of Inflammation. Nature.

[5] Hotchkiss R.S., Karl I.E. (2003). The Pathophysiology and Treatment of Sepsis. N. Engl. J. Med..

[6] Vigata M., Meinert C., Hutmacher D.W., Bock N. (2020). Hydrogels as Drug Delivery Systems: A Review of Current Characterization and Evaluation Techniques. Pharmaceutics.

[7] Qu J., Zhao X., Liang Y., Xu Y., Ma P.X., Guo B. (2019). Degradable Conductive Injectable Hydrogels as Novel Antibacterial, Anti-Oxidant Wound Dressings for Wound Healing. Chem. Eng. J..

[8] Ogay V., Mun E.A., Kudaibergen G., Baidarbekov M., Kassymbek K., Zharkinbekov Z., Saparov A. (2020). Progress and Prospects of Polymer-Based Drug Delivery Systems for Bone Tissue Regeneration. Polymers.

[9] Wang H., Feng Y., Lu H. (2022). Low-Level Cefepime Exposure Induces High-Level Resistance in Environmental Bacteria: Molecular Mechanism and Evolutionary Dynamics. Environ. Sci. Technol..

[10] Shi Q., Luo X., Huang Z., Midgley A.C., Wang B., Liu R., Zhi D., Wei T., Zhou X., Qiao M. (2019). Cobalt-Mediated Multi-Functional Dressings Promote Bacteria-Infected Wound Healing. Acta Biomater..

[11] Re’em T., Witte F., Willbold E., Ruvinov E., Cohen S. (2012). Simultaneous Regeneration of Articular Cartilage and Subchondral Bone Induced by Spatially Presented TGF-Beta and BMP-4 in a Bilayer Affinity Binding System. Acta Biomater..

[12] Chen K., Wang F., Liu S., Wu X., Xu L., Zhang D. (2020). In Situ Reduction of Silver Nanoparticles by Sodium Alginate to Obtain Silver-Loaded Composite Wound Dressing with Enhanced Mechanical and Antimicrobial Property. Int. J. Biol. Macromol..

[13] Diniz F.R., Maia R.C.A.P., de Andrade L.R.M., Andrade L.N., Vinicius Chaud M., da Silva C.F., Corrêa C.B., de Albuquerque Junior R.L.C., Pereira da Costa L., Shin S.R. (2020). Silver Nanoparticles-Composing Alginate/Gelatine Hydrogel Improves Wound Healing In Vivo. Nanomaterials.

[14] Lin X., Guan X., Wu Y., Zhuang S., Wu Y., Du L., Zhao J., Rong J., Zhao J., Tu M. (2020). An Alginate/Poly (N-Isopropylacrylamide)-Based Composite Hydrogel Dressing with Stepwise Delivery of Drug and Growth Factor for Wound Repair. Mater. Sci. Eng. C.

[15] Zhang M., Zhao X. (2020). Alginate Hydrogel Dressings for Advanced Wound Management. Int. J. Biol. Macromol..

[16] Aderibigbe B.A., Buyana B. (2018). Alginate in Wound Dressings. Pharmaceutics.

[17] Boateng J., Catanzano O. (2015). Advanced Therapeutic Dressings for Effective Wound Healing—A Review. J. Pharm. Sci..

[18] Yang K., Han Q., Chen B., Zheng Y., Zhang K., Li Q., Wang J. (2018). Antimicrobial Hydrogels: Promising Materials for Medical Application. Int. J. Nanomed..

[19] Zhang H., Peng M., Cheng T., Zhao P., Qiu L., Zhou J., Lu G., Chen J. (2018). Silver Nanoparticles-Doped Collagen–Alginate Antimicrobial Biocomposite as Potential Wound Dressing. J. Mater. Sci..

[20] Alshammari F., Alshammari B., Moin A., Alamri A., Al Hagbani T., Alobaida A., Baker A., Khan S., Rizvi S.M.D. (2021). Ceftriaxone Mediated Synthesized Gold Nanoparticles: A Nano-Therapeutic Tool to Target Bacterial Resistance. Pharmaceutics.

[21] Farhadian N., Karimi M., Porozan S. (2021). Ceftriaxone Sodium Loaded onto Polymer-Lipid Hybrid Nanoparticles Enhances Antibacterial Effect on Gram-Negative and Gram-Positive Bacteria: Effects of Lipid-Polymer Ratio on Particles Size, Characteristics, In Vitro Drug Release and Antibacterial Drug Ef. J. Drug Deliv. Sci. Technol..

[22] Adil M., Alam S., Amin U., Ullah I., Muhammad M., Ullah M., Rehman A., Khan T. (2023). Efficient Green Silver Nanoparticles-Antibiotic Combinations against Antibiotic-Resistant Bacteria. AMB Express.

[23] Han S.-K. (2023). Innovations and Advances in Wound Healing.

[24] Dolati S., Yousefi M., Pishgahi A., Nourbakhsh S., Pourabbas B., Shakouri S.K. (2020). Prospects for the Application of Growth Factors in Wound Healing. Growth Factors.

[25] Kim S.-W., Im G.-B., Jeong G.-J., Baik S., Hyun J., Kim Y.-J., Pang C., Jang Y.C., Bhang S.H. (2021). Delivery of a Spheroids-Incorporated Human Dermal Fibroblast Sheet Increases Angiogenesis and M2 Polarization for Wound Healing. Biomaterials.

[26] Kuhn L.T., Peng T., Gronowicz G., Hurley M.M. (2021). Endogenous FGF-2 Levels Impact FGF-2/BMP-2 Growth Factor Delivery Dosing in Aged Murine Calvarial Bone Defects. J. Biomed. Mater. Res. Part A.

[27] Dinh T., Braunagel S., Rosenblum B.I. (2015). Growth Factors in Wound Healing: The Present and the Future?. Clin. Podiatr. Med. Surg..

[28] Hayes A.J., Whitelock J., Melrose J. (2022). Regulation of FGF-2, FGF-18 and Transcription Factor Activity by Perlecan in the Maturational Development of Transitional Rudiment and Growth Plate Cartilages and in the Maintenance of Permanent Cartilage Homeostasis. Int. J. Mol. Sci..

[29] Novais A., Chatzopoulou E., Chaussain C., Gorin C. (2021). The Potential of FGF-2 in Craniofacial Bone Tissue Engineering: A Review. Cells.

[30] Abayev U. (2003). Wound Dressings in Surgery. Med. News.

[31] Akhmetkarimova Z.S., Kudaibergen G.K., Kaukabaeva G.K., Abeldenov S.K., Rysbek A.B. (2023). Thiol-Ene Click Synthesis of Alginate Hydrogels Loaded with Silver Nanoparticles and Cefepime. Eurasian J. Chem..

[32] Zafar N., Shamaila S., Nazir J., Sharif R., Shahid Rafique M., Ul-Hasan J., Ammara S., Khalid H. (2016). Antibacterial Action of Chemically Synthesized and Laser Generated Silver Nanoparticles against Human Pathogenic Bacteria. J. Mater. Sci. Technol..

[33] Kudaibergen G., Akhmetkarimova Z., Yildirim E., Baidarbekov M. (2023). Thiol-Ene Clickable Gelatin–Hyaluronic Acid Cryogels. J. Mater. Sci..

[34] (2009). Tests for In Vitro Cytotoxicity.

[35] Balouiri M., Sadiki M., Ibnsouda S.K. (2016). Methods for In Vitro Evaluating Antimicrobial Activity: A Review. J. Pharm. Anal..

[36] Adamiak K., Sionkowska A. (2023). State of Innovation in Alginate-Based Materials. Mar. Drugs.

[37] Rosiak P., Latanska I., Paul P., Sujka W., Kolesinska B. (2021). Modification of Alginates to Modulate Their Physic-Chemical Properties and Obtain Biomaterials with Different Functional Properties. Molecules.

[38] Bruna T., Maldonado-Bravo F., Jara P., Caro N. (2021). Silver Nanoparticles and Their Antibacterial Applications. Int. J. Mol. Sci..

[39] Grambow E., Sorg H., Sorg C.G.G., Strüder D. (2021). Experimental Models to Study Skin Wound Healing with a Focus on Angiogenesis. Med. Sci..

